# Long-term outcomes and temporal trends following liver transplantation for chronic liver disease in the intensive care unit

**DOI:** 10.1016/j.jhepr.2025.101722

**Published:** 2026-01-08

**Authors:** Magdalena Meszaros, José Ursic-Bedoya, Audrey Coilly, Claire Francoz, Cristophe Duvoux, Filomena Conti, Francois Faitot, Pauline Houssel-Debry, Jean Hardwigsen, Marie-Noelle Hilleret, Claire Vanlemmens, Laure Elkrief, Nassim Kamar, Rodolphe Anty, Armand Abergel, Claire Perignon, Laurence Chiche, Maryline Debette-Gratien, Teresa Antonini, Corinne Antoine, Sebastien Dharancy, Jérome Dumortier, Georges Philippe Pageaux, Florent Artru

**Affiliations:** 1 Liver Transplant and Hepatogastroenterology Unit, CHU Saint-Eloi, Montpellier University, Montpellier, France; 2 Hepatobiliary Center, AP-HP Paul Brousse Hospital, Villejuif, France; 3 Hepatology Unit, Beaujon Hospital, Assistance Publique-Hôpitaux de Paris, Clichy, France; 4 Hepatology and Medical Liver Transplant Unit, Henri-Mondor Hospital-APHP, Paris Est University Créteil, France; 5 Hepatology and Liver Transplant Unit, AP-HP, Groupe Hospitalier Pitié-Salpêtrière, Paris, France; 6 Liver Transplant and Digestive Surgery Unit, Strasbourg University, Strasbourg, France; 7 Liver Department, Rennes University Hospital and Rennes University, UMR 1317 NuMeCan Institute and REnnes LIvEr Failure Group (RELIEF), France; 8 Hepatogastroenterology Unit, CHU Timone, Marseille, France; 9 Université Grenoble Alpes, Service Hépatogastroenterologie CHUGrenoble Alpes, Inserm U 1209, CNRS UMR 5309, Team Cell Dynamics, Immunity, Metabolism & Cancer, IAB 38000 Grenoble, France; 10 Hepatology Unit, CHRU Jean Minjoz Franche Comté University, Besançon, France; 11 Hepatogastroenterology Unit, CHU Tours, Tours, France; 12 Department of Nephrology and Organ Transplantation, Axe TImE, Toulouse Rangueil University Hospital, INSERM UMR 1291, Toulouse Institute for Infectious and Inflammatory Diseases (Infinity), University Paul Sabatier, Toulouse, France; 13 Hepatogastroenterology Unit, Nice University, France; 14 Hepatogastroenterology Unit, CHU Estaing Clermont-Ferrand, Clermont-Ferrand, France; 15 Department of Hepato-Gastroenterology, CHU Côte de Nacre, Caen, France; 16 Hepatogastroenterology Unit, Haut Leveque Hospital, CHU Bordeaux, Bordeaux, France; 17 Hepatogastroenterology Unit, CHU Limoges, Limoges, France; 18 Hepatology Unit, HCL, Hôpital de la Croix-Rousse, Lyon, France; 19 Direction Générale Médicale et Scientifique, Agence de la Biomédecine, Saint Denis, France; 20 Hepatology Unit, CHRU Lille, Claude Huriez Hospital, Lille, France; 21 Liver Transplant Unit, Digestive Diseases Federation, Edouard Herriot Hospital, Hospices Civils de Lyon Université, Lyon, France

**Keywords:** liver transplantation, intensive care unit, long-term mortality

## Abstract

**Background & Aims:**

Liver transplantation (LT) remains the definitive treatment for patients with end-stage chronic liver disease (CLD). However, those transplanted while in the intensive care unit (ICU) represent a high-risk population. Large-scale data on long-term prognosis in this group are limited. We aimed to assess long-term outcomes in patients with CLD undergoing LT from the ICU and to compare outcomes over time.

**Methods:**

This retrospective cohort study used the French national transplant registry (CRISTAL). Adults with CLD who underwent LT from the ICU between 2008 and 2018 were included. Organ failures were defined according to EASL CLIF-OF criteria. Five-year survival and associated risk factors were analyzed and compared across four time periods (2008–2010, 2011–2013, 2014–2016, 2017–2018).

**Results:**

Among 13,372 LTs performed in France during the study period, 9,686 were for CLD, of which 1,287 (13.2%) patients were in the ICU at the time of LT. Alcohol-related liver disease (50%) and viral hepatitis (16.1%) were the leading etiologies. Five-year survival was significantly lower in ICU patients compared with non-ICU patients (69.2% *vs.* 79.1%, *p <*0.0001). Among patients who survived the first post-transplant year, 5-year survival exceeded 83% and was comparable to that of patients with CLD transplanted outside the ICU. Survival by era showed no significant improvement (*p =* 0.28). Age (hazard ratio [HR] 1.03, *p <*0.0001), mechanical ventilation (HR 1.57, *p =* 0.0001) and French donor risk score (HR 1.05, 95% CI [1.02-1.09], *p <*0.001) were independent predictors of mortality.

**Conclusion:**

Patients transplanted from the ICU have significantly lower long-term survival, primarily due to elevated early post-transplant mortality, with no observed improvement over time. Careful candidate evaluation and donor selection remain critical to improving outcomes in this high-risk population.

ClinicalTrials.gov number NCT06636409.

**Impact and implications:**

This large national cohort study provides a comprehensive evaluation of long-term outcomes in critically ill patients with cirrhosis undergoing liver transplantation while in the intensive care unit. Despite advances in transplant care over the past decade, we observed a persistent survival gap in this high-risk population, primarily driven by increased mortality within the first year after transplantation. Age, the need for mechanical ventilation, and donor-related factors were independently associated with this excess risk. We also found no significant improvement in outcomes over time. These results underscore the continued need for refined candidate selection and donor allocation strategies, taking into account age, pre-transplant clinical stability, and graft quality to optimize post-transplant survival.

## Highlights

• Patients with CLD transplanted from the ICU represent a particularly high-risk liver transplant population.
• In 1,287 ICU recipients, 5-year survival was lower than in non-ICU recipients (69% *vs.* 79%).
• Among 1-year survivors, long-term survival was comparable between ICU and non-ICU groups.
• Long-term outcomes did not improve over time despite increased ICU transplant activity.
• Older age, mechanical ventilation, and donor risk independently predicted 5-year mortality.

## Graphical abstract

**Figure:**
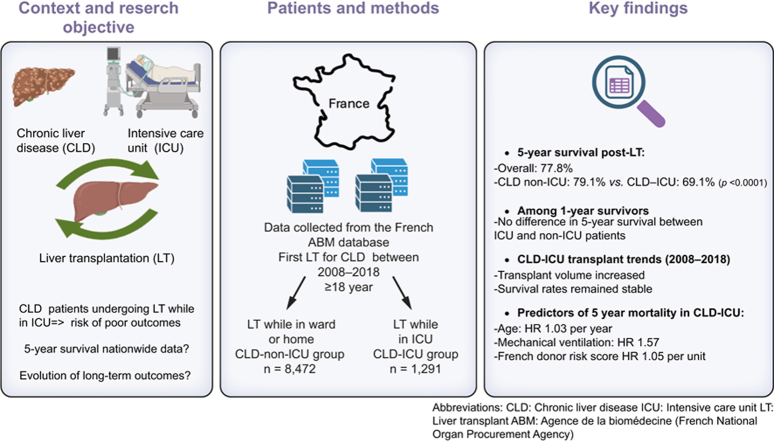

## Introduction

Liver transplantation (LT) is the only curative option for patients with end-stage chronic liver disease (CLD) and unresectable hepatocellular carcinoma1.^1^ In recent years, notable progress in immunosuppressive strategies, surgical approaches, and the prevention of post-operative complications has significantly enhanced patient management.^2^ Moreover, the prognosis of certain causes of cirrhosis, such as HCV-related cirrhosis, has improved markedly both before and after LT, following the advent of effective treatments such as direct-acting antivirals.^3^ Hence, over the past 30 years, major progress in 1-year survival has been observed globally.^1^^,^^2^^,^[4], [5], [6] For instance, in France, 1-year survival rates increased from 78% to 89%, between the 90s and the 2020s.^7^ In parallel with the gains in short-term post-transplant survival, long-term outcomes have also shown steady progress, with large retrospective registries reporting 5- and 10-year survival rates of approximately 80% and 60–70%, respectively.^5^

Among patients with CLD undergoing LT, those transplanted while hospitalized in the intensive care unit (CLD-ICU) represent a particularly high-risk group.^8^ Indeed, these patients frequently present with multi-organ failure and require organ support, such as mechanical ventilation or renal replacement therapy (RRT), which are considered predictors of poor outcomes.[9], [10], [11] However, a growing body of evidence suggests that LT is feasible and can achieve favorable short-term outcomes in carefully selected patients with decompensated cirrhosis in the ICU. Although little is known about their long-term outcomes.^12^^,^^13^ A multicenter retrospective study of 73 patients with acute-on-chronic liver failure (ACLF) grade 3 across three centers found that LT could achieve favorable long-term outcomes, meeting the commonly accepted utility threshold of 50–70% 5-year post-transplant survival.^13^^,^^14^ However, whether the long-term results, derived mainly from high-volume centers, are observed at a nationwide level, remains to be demonstrated.

Additionally, recent data indicate that in this specific population, a learning curve in patient selection and bridging strategies to transplantation plays a critical role in achieving favorable outcomes. As an example, in the US, over a period of 16 years, the 1-year survival rate for patients transplanted while in the ICU has improved over time, increasing from 72.5% in 2005–2008 to 89.5% in 2017–2020.^15^ It remains unclear whether similar improvements have occurred in other countries or whether they are sustained over the long term.

Thus, in the present study, we aimed to evaluate, at the national level in France, the long-term survival of patients with CLD who underwent LT while in the ICU, and to assess whether, as observed in the US, survival trends have improved over time and at long-term follow-up.

## Patients and methods

### Study design

This was a retrospective cohort study using prospectively collected data from the CRISTAL national transplant database, supported by the French organ procurement agency (Agence de la Biomédecine-ABM). The study was approved by the Scientific Committee of the ABM and by the ethical committee of the University Hospital of Montpellier (2019-IRB-MTP_09-13). The requirement for informed consent was waived in accordance with the French “Jardé” law governing clinical research due to the retrospective, non-interventional nature of the study, which utilized anonymized data. This research was conducted in accordance with both the Declarations of Helsinki and Istanbul.

### Study population

All adult patients (aged ≥18 years) with CLD who underwent a first LT in France between January 1^st^ 2008 and December 31^st^ 2018 were eligible for inclusion. Patients were excluded if they were transplanted for acute liver failure, underwent re-LT, or if the indication for LT was secondary liver malignancies, benign tumors or a liver disease of unknown etiology. Based on data extracted from the CRISTAL database, we harmonized various criteria under the definition of CLD, as defined in EASL guidelines,^16^ including cirrhosis, advanced chronic liver disease, and related classifications. This study population was divided into two groups: those transplanted while hospitalized in the ICU (CLD-ICU) and those transplanted from a ward or from home (CLD-non-ICU). To investigate survival trends over time, the outcomes were further stratified into four time periods: 2008–2010, 2011–2013, 2014–2016, and 2017–2018.

### Data collection

All the following data were extracted from the prospectively collected CRISTAL database: demographic characteristics, liver disease etiology, indication for LT, biological parameters such as creatinine, prothrombin time, bilirubin, MELD (model for end-stage liver disease) score, site of hospitalization at time of LT (ICU, ward or from home), and use of mechanical ventilation or RRT at LT, as well as donor data. Evaluation of graft quality was estimated by the French donor risk score. This score was developed by the ABM and estimates the 1-year graft failure risk based on donor characteristics. It incorporates donor age over 65, history of hypertension or vascular cause of death, diabetes, and significant donor-recipient height mismatch. The formula is as follows 0.16193 × (age >65) + 0.30218 × (HTA/vascular death) + 0.23453 × (diabetes) + 0.23931 × (height mismatch) and each variable is binary (1 = present, 0 = absent). This score was selected because the Eurotransplant and American donor risk indexes have previously been shown to perform poorly in the French liver donor context, likely due to significant differences in donor and recipient characteristics across transplant systems.^17^ While the CRISTAL database includes assessments of hepatic, renal, and coagulation failures, it lacks data on PaO_2_/FiO_2_ ratios for respiratory evaluation, as well as information on neurological status and vasopressor use. Post-transplant outcomes such as last follow-up visit, causes and date of death were also extracted from the CRISTAL database, up to August 2024.

### Definitions

Organ failure was defined according to the EASL CLIF-OF criteria.^18^ Liver failure was defined by a bilirubin level ≥205 μmol/L, kidney failure was defined by a serum creatinine level ≥176.8 μmol/L or the need for RRT, coagulation failure was defined by an international normalized ratio ≥2.5. For respiratory failure, as there is no information on PaO_2_/FiO_2_ ratio ≤200 in the CRISTAL database, we considered need for mechanical ventilation as a marker of respiratory failure as done in other registry-based work.^19^^,^^20^ Neurological failure and circulatory status could not be assessed, therefore a precise ACLF staging per CLIF criteria was not feasible.

### Statistical analyses

Continuous variables were expressed as means ± SD or medians (IQR) and compared using the Student’s *t* test or Mann-Whitney *U* test as appropriate. Categorical variables were expressed as frequencies (percentages) and compared using the Chi-square test. The primary endpoint was 5-year survival following LT. Secondary outcomes included temporal trends of mortality over the four time periods, causes of death, predictors of 1- and 5-year mortality. Kaplan-Meier survival curves were generated to assess 1- and 5-year survival, and differences between groups were tested using the log-rank test. A multivariate Cox proportional hazards model was used to identify independent predictors of mortality. Data were checked for multicollinearity with the Belsley-Kuh-Welsch technique and proportional hazards were checked according to Schoenfeld residuals. The alpha risk was set to 5%. A *p* value of <0.05 was considered statistically significant. Data were analyzed using EasyMedStat (version 3.0).

## Results

### Baseline characteristics

**Fig. 1:**
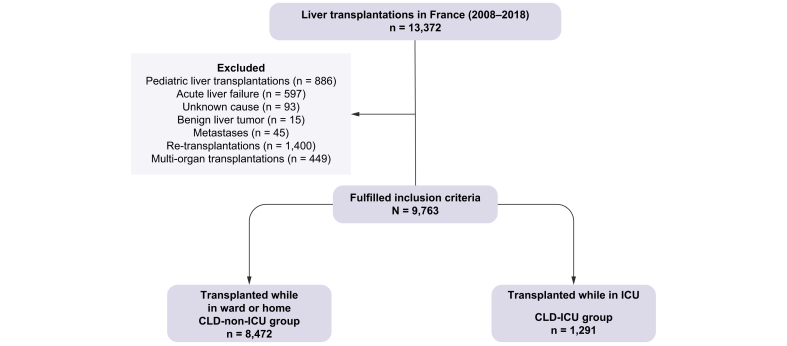
Study flowchart of patients identified through CRISTAL database. CLD, chronic liver disease; ICU, intensive care unit.

Between 2008 and 2018, a total of 13,372 liver transplants were performed in France, of which 9,763 were in patients with CLD (Fig. 1). Their characteristics are shown in Table S1. A total of 1,291 patients (13.2% of the overall cohort with CLD) were transplanted while in the ICU (CLD-ICU). Compared to CLD non-ICU patients, these patients were younger (53.3 years (±10.5) *vs.* 55.2 years ± 9.9; *p <*0.001) and had a higher MELD score (28.1 (±9.8) *vs.* 13.5 (±8.8), *p <*0.001).

In the CLD-ICU cohort, alcohol-related liver disease (n = 645, 49.9%) and cirrhosis due to viral hepatitis (n = 208, 16.1%) were the most common causes of underlying disease. 1,201 patients (93.0%) were primarily listed for decompensated cirrhosis and 90 (7.0%) for hepatocellular carcinoma. At LT, 403 patients (34.1%) in the CLD-ICU group required mechanical ventilation, and 289 (22.9%) were under RRT. As for donor characteristics, the overall mean donor age was 55.8 ± 18.7 years in the CLD non-ICU group compared to 57.6 ± 17.5 years in the CLD-ICU group (*p* <0.001). The French donor risk score was 2.55 ± 2.88 in the CLD non-ICU group and 2.75 ± 2.90 in the CLD-ICU group (*p =* 0.01) Table 1.

**Table 1:** Baseline characteristics of patients with CLD according to ICU status at the time of LT.

Characteristics of patients	CLD non-ICU n = 8,472	CLD-ICU n = 1,291	*p* value
Sex (male)	6,503 (76.7%)	915 (70.8%)	<0.001
Age at LT (years), mean (SD)	55.2 (±9.9)	53.25 (±10.5)	<0.001
BMI (kg/m^2^)	26.6 (±6.2)	26.3 (±5.3)	0.07
Time period of LT			0.001
2008-2010	2,037 (91.4%)	192 (8.6%)	
2011-2013	2,187 (85.1%)	384 (14.9%)	
2014-2016	2,563 (85.8%)	424 (14.2%)	
2017-2018	1,685 (85.4%)	291 (14.7%)	
Initial disease requiring LT, n (%)			<0.001
ALD	2,566 (30.3%)	645 (50%)	
Viral	950 (11.2%)	208 (16.1%)	
Auto-immune, cholestatic	716 (8.4%)	148 (11.5%)	
Others	4,240 (50%)	290 (22.5%)	
HCC status as initial disease			<0.001
HCC	3,294 (38.9%)	90 (7%)	
No HCC	5,178 (61.1%)	1,201 (93%)	
Biological parameters at LT			
Bilirubin (μmol/L), mean (SD)	84.8 (±124.8)	333.3 (±304.3)	<0.001
Creatinine (μmol/L), mean (SD)	81 (±36.7)	119.82 (±82.6)	<0.001
Sodium (mmol/L), mean (SD)	136.4 (±5.3)	136.92 (±6.2)	0.11
INR, mean (SD)	1.8 (±1)	2.9 (±2)	<0.001
MELD score at LT, median (IQR)	12.27 (±12.37)	27.49 (±13.19)	<0.001
MELD 3.0 score at LT, median (IQR)	19 (±11.7)	31.8 (±13.3)	<0.001
Mechanical ventilation at LT	84 (1%)	403 (34%)	<0.001
Renal replacement therapy	44 (0.5%)	289 (22.9%)	<0.001
Liver failure	907 (10.7%)	810 (62.7%)	<0.001
Renal failure	252 (3%)	482 (37.3%)	<0.001
Coagulation failure	1,085 (12.8%)	573 (44.4%)	<0.001
Number of organ failures			
1	1,444 (17%)	287 (22.2%)	<0.001
2	470 (5.5%)	332 (25.7%)	
≥3	50 (0.6%)	434 (33.6%)	
Liver graft parameters			
Donor age (years), Mean (SD)	55.8 (±18.7)	57.62 (±17.53)	<0.001
Cause of death cardiovascular	4,874 (57.5%)	807 (62.5%)	0.04
Female sex donor	3,787 (44.7%)	618 (47.8%)	0.03
French donor risk score∗	2.55 (±2.88)	2.75 (±2.9)	0.01
Donor risk score ≥6	1,317 (15.5%)	207 (16%)	0.8

ALD, alcohol-related liver disease; CLD, chronic liver disease; HCC, hepatocellular carcinoma; ICU, intensive care unit; INR, international normalized ratio; LT, liver transplantation; MELD, model for end-stage liver disease; OF, organ failure.

Data are expressed as number (%) or as mean ± standard deviation and median [Q1-Q3]. *p* values are the results of chi^2^ test for qualitative variables or *t* test for quantitative variables. *p* <0.05 is significant.

∗ The French donor risk score is defined by 0.16193 × (age >65) + 0.30218 × (HTA/vascular death) + 0.23453 × (diabetes) + 0.23931 × (height mismatch) Each variable is binary (1 = present, 0 = absent).

The mean overall follow-up in the entire cohort was of 6.9 (±4.3) years.

### Outcomes at 5 years

**Fig. 2:**
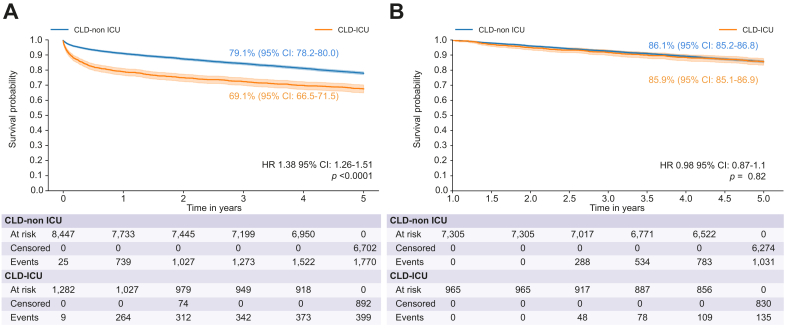
Survival probability by ICU status. (A) 5-year survival of patients with CLD according to ICU status at LT. (B) 5-year survival after LT among 1-year survivors (CLD non-ICU *vs*. CLD-ICU). CLD, chronic liver disease; ICU, intensive care unit; LT, liver transplantation. Kaplan-Meyer and Log-rank test were used, and significant was considered if *p* <0.005.

**Fig. 3:**
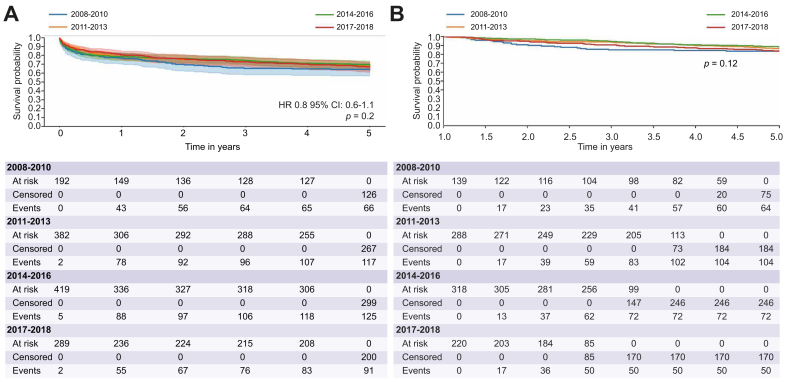
Survival probability by time period of LT. (A) 5-year survival probability in CLD-ICU patients according to time period of LT. (B) 5-year survival probability in CLD-ICU patients among 1-year survivors according to time period of LT. CLD, chronic liver disease; ICU, intensive care unit; LT, liver transplantation. Kaplan-Meyer test and Log-Rank test were used, significant if *p* <0.05.

The overall 5-year survival rate for all patients with CLD who underwent LT was 77.8% (95% CI 76.9-78.6) and differed between the CLD non-ICU (79.1%; 95% CI 78.2-80.0) and CLD-ICU (69.1%; 95% CI 66.5-71.5; *p <*0.0001) groups (hazard ratio [HR] 1.38; 95% CI 1.26-1.51; *p <*0.0001) (Fig. 2). These survival estimates were based on the occurrence of 3,050 (36.0%) and 554 (42.9%) deaths in the CLD non-ICU and CLD-ICU groups, respectively (*p <*0.0001). Median time to death in both groups was 1,389 (±1,418) days *vs.* 467 days (±1,382, *p =* 0.001), respectively. Specifically, in the CLD-ICU group, 264 (47.8%) deaths occurred within 1 year after LT and 312 (56.3 %) within the first 2 years. Specific causes of early (≤3 months) death for the 170 (30.7%) patients in CLD-ICU group are given in Table S2. In patients alive after 1 year, we observed an absence of difference in 5-year survival between CLD-ICU and CLD non-ICU groups (86.0% *vs.* 85.9%, *p =* 0.4) (Fig. 3).

The main causes of 5-year mortality post-LT differed between the groups. Infections (22.7% *vs.* 12.4%, *p <*0.001) and cardiovascular events (12.8% *vs.* 9.7%, *p <*0.001) were more common in the CLD-ICU group, whereas cancer (17.0% *vs.* 9.4%,) and liver graft-related deaths (17.8% *vs.* 10.3%, *p <*0.001) were more frequent in the CLD non-ICU group, as shown in Table 2. In multivariate analysis of the entire cohort, ICU status was the only variable independently associated with infection-related mortality (odds ratio = 1.55; 95% CI 1.05–2.29; *p* = 0.02), whereas age, dialysis, intubation, and bilirubin were not. Similarly, for cardiovascular-related mortality, only ICU status remained independently associated (odds ratio = 1.68; 95% CI 1.1–2.5; *p* = 0.01).

**Table 2:** Causes of 5-year mortality after LT according to ICU status at LT.

Causes of death	CLD non-ICU (n = 3,050)	CLD-ICU (n = 554)	*p* value
Infections, n (%)	378 (12.4)	126 (22.7)	<0.0001
Cardiovascular event, n (%)	295 (9.7)	71 (12.8)	
Cancer, n (%)	517 (17)	52 (9.4)	
Other, n (%)	1,279 (41.9)	237 (42.8)	
Liver graft related, n (%)	545 (17.9)	57 (10.3)	

CLD, chronic liver disease; ICU, intensive care unit; LT, liver transplantation.

Data are expressed as number (%), *p* values are the results of chi^2^ test for qualitative variables *p* <0.05 is significant.

As for graft survival, the overall 5-year graft survival rate for all CLD recipients who underwent LT was 71.4% (95% CI 70.5-72.3) and differed between the CLD non-ICU (72.6%; 95% CI 71.5-73.4) and CLD-ICU (63.6%; 95% CI 60.9-66.2; *p <*0.0001) groups (HR 1.29; 95% CI 1.18-1.4; *p <*0.0001) (Fig. S1).

### 5-year outcome trends over time

The percentage of patients transplanted while in the ICU increased over time from 8.6% of the CLD population in 2008-2010, to 14.9% in 2011-2013, to 14.2% in 2014-2016 and 14.7% in 2017-2018 (*p <*0.001). The evolution of patient characteristics over time is presented in Table 3 and Table S3. Specifically, the proportion of patients transplanted with HCV decreased from 16.2% to 5.5% (*p <*0.001), and the proportion of patients transplanted while on mechanical ventilation decreased from 42.2% to 28.0% (*p <*0.001), while sex, age or dialysis remained non-different over time. As for donor characteristics for the CLD-ICU group, mean age rose from 53.63 ± 16.71 years in 2008–2012 to 59.65 ± 17.33 years in 2017–2018 (*p* = 0.001). Causes and evolution of 5 year mortality in CLD-ICU patients according to time period is represented in Table 4.

**Table 3:** Temporal trends in characteristics of CLD-ICU patients at the time of LT.

Variable	2008-2010 n = 192	2011-2013 n = 384	2014-2016 n = 424	2017-2018 n = 291	*p* value
Sex male, (%)	125 (65.1%)	275 (71.6%)	306 (72.2%)	209 (71.8%)	0.3
Age at LT (years), mean (SD)	53.4 (±9.9)	53 (±10.1)	53 (±11.1)	53.9 (±10.6)	0.43
Initial disease requiring LT, n (%)					0.001
ALD	82 (42.7%)	188 (49%)	220 (51.9%)	164 (56.4%)	
Viral B/C	8 (4.2%)/31 (16.2%)	20 (5.2%)/63 (16.4%)	20 (4.7%)/38 (9%)	12 (4.1%)/16 (6%)	
Autoimmune, cholestatic	29 (15.1%)	43 (11.2%)	45 (10.61%)	31 (10.65%)	
Others	25 (13%)	44 (11.5%)	65 (15.3%)	55 (18.9%)	
HCC status as initial disease					
HCC	17 (8.8%)	26 (6.8%)	36 (8.5%)	17 (8.8%)	
Biological parameters at LT					
Creatinine (μmol/L), mean (SD)	114.1 (±72.8)	120.7 (±80.3)	121.5 (±90)	119.9 (±81.5)	0.86
Bilirubin (μmol/L), mean (SD)	365.3 (±247.7)	334.31 (±220.8)	325.19 (±422.5)	321.48 (±211.3)	0.08
INR, mean (SD)	2.7 (±1.4)	3 (±2.5)	3 (±1.9)	2.8 (±1.4)	0.9
MELD score at LT, mean (SD)	28.32 (±9.6)	28.5 (±9.7)	27.52 (±10.4)	28.15 (±9.2)	0.5
Mechanical ventilation at LT	70 (42.2%)	135 (40.5%)	119 (30.7%)	79 (28%)	0.001
Renal replacement therapy	49 (27.1%)	67 (18%)	103 (24.6%)	70 (24.1%)	0.05
Liver failure	125 (65.1%)	247 (64.3%)	255 (60.1%)	183 (62.9%)	0.55
Renal failure	72 (37.5%)	131 (34.1%)	165 (38.9%)	114 (39.2%)	0.46
Coagulation failure	85 (44.3%)	182 (47.4%)	181 (42.7%)	125 (43%)	0.54
Liver graft parameters					
Donor age (years), mean (SD)	53.63 (±16.71)	56.55 (±17.77)	59.01 (±17.5)	59.65 (±17.33)	0.001
Cause of death cardiovascular n (%)	121 (62.7%)	238 (61.8%)	265 (62.5%)	183 (63.1%)	0.06
Female sex donor	83 (43%)	184 (47.7%)	206 (48.6%)	145 (49.8%)	0.5
French donor risk score∗	2.03 (±2.73)	2.6 (±2.88)	2.98 (±2.91)	3.09 (±2.92)	0.001
Donor risk score ≥6	15 (7.77%)	60 (15.54%)	77 (18.16%)	55 (18.9%)	0.004

ALD, alcohol-related liver disease; HCC, hepatocellular carcinoma; INR, international normalized ratio; ICU, intensive care unit; LT, liver transplantation; MELD, model for end-stage liver disease; RRT, renal replacement therapy. The French donor risk score is defined by 0.16193 × (age >65) + 0.30218 × (HTA/vascular death) + 0.23453 × (diabetes) + 0.23931 × (height mismatch). Each variable is binary (1 = present, 0 = absent).Data are expressed as number (%) or as mean ± standard deviation and median [Q1-Q3]. *p* values are the results of chi^2^ test for qualitative variables or *t* test for quantitative variables. *p* <0.05 is significant.

**Table 4:** Causes of 5-year mortality after LT in CLD-ICU patients by time period.

Causes of death	2008-2010	2011-2013	2014-2016	2017-2018	*p* value
Infections, n (%)	23 (21.5%)	32 (17.6%)	42 (26.2%)	29 (27.6%)	0.442
Cardiovascular event, n (%)	13 (12.1%)	24 (13.2%)	19 (11.9%)	14 (13.3%)	
Cancer, n (%)	15 (14%)	13 (7.1%)	12 (7.5%)	11 (10.5%)	
Other, n (%)	45 (42.1%)	95 (52.2%)	69 (43.1%)	41 (39.1%)	
Liver graft-related, n (%)	11 (10.3%)	18 (9.9%)	18 (11.2%)	10 (9.5%)	

CLD, chronic liver disease; HR, hazard ratio; ICU, intensive care unit; LT, liver transplantation; MELD, model for end-stage liver disease.

ICU, intensive care unit.

Data are expressed as number (%). *p* values are the results of chi^2^ test for qualitative variables *p* <0.05 is significant.

Five-year survival rates for LT recipients were then further analyzed in four time periods (2008–2010 *vs.* 2011–2013 *vs.* 2014–2016 *vs.* 2017–2018). In the overall CLD cohort, 5-year survival rates were 75.7% (95% CI 73.9%–77.5%) in 2008–2010, 79.3% (95% CI 77.6%–80.8%) in 2011–2013, 77.5% (95% CI 76.0%–79.0%) in 2014–2016, and 78.5% (95% CI 76.7%–80.3%) in 2017–2018 (*p* = 0.0186). Specifically, from 2008-2010 to 2017-2018, 5-year survival rates improved from 75.7% (95% CI 73.9%–77.5%) to 78.5% (95% CI 76.7%–80.3%) (*p* = 0.01) (Fig. S2). In patients alive after 1 year, 5-year survival rates were 87.3% *vs.* 87.3% *vs.* 87.8% *vs.* 85.5% (*p =* 0.13), respectively.

We further performed sensitivity analyses restricted to CLD-ICU and CLD non-ICU cohorts. In the CLD-ICU cohort, 5-year survival rates were 65.6% (95% CI 58.4%–71.9%) in 2008–2010, 69.5% (95% CI 64.7%–73.9%) in 2011–2013, 70.5% (95% CI 65.9%–74.6%) in 2014–2016, and 68.7% (95% CI 63.1%–73.7%) in 2017–2018 (*p =* 0.2) (Fig. 3A). Although a numerical increase in 5-year survival was observed from 2008–2010 to 2017–2018, this difference was not statistically significant. Moreover, when we restricted the analysis in the CLD-ICU group to two time periods (2008-2013 *vs.* 2014-2018) we did not see different long-term outcomes (Fig. S4). In CLD-ICU patients alive after 1 year, 5-year survival rates were 83.5% (95% CI 76.2%–88.7%) in 2008–2010, 86.5% (95% CI 81.9%–89.9%) in 2011–2013, 88.4% (95% CI 84.3%–91.4%) in 2014–2016, and 83.6% (95% CI 78.0%–87.9%) in 2017–2018 (*p =* 0.12) (Fig. 3B).

In CLD non-ICU patients, 5-year survival rates were 77.5% (95% CI 75.6%–79.3%) in 2008-2010, 81.2% (95% CI 79.5%–82.8%) in 2011-2013, 78.8% (95% CI 77.2%–80.4%) in 2014-2016 and 80.3% (95% CI 78.3%–82.1 %) in 2017-2018 (Fig. S3). Specifically, from 2008-2010 to 2017-2018, 5-year survival rates improved from 77.5% to 80.3% (*p* = 0.02).

### Factors associated with 1-year and 5-year mortality

In the overall population, factors associated with 5-year mortality in univariable analysis included age at LT (HR 1.03; 95% CI 1.03–1.03; *p <*0.0001), mechanical ventilation (HR 1.67; 95% CI 1.46–1.90; *p <*0.0001), RRT (HR 1.59; 95% CI 1.35–1.86; *p <*0.0001) and ICU status at LT (HR 1.38; 95% CI 1.26–1.51; *p <*0.0001). In multivariable cox regression, risk factors for 5-year mortality after LT were age (HR 1.03; 95% CI 1.03–1.04; *p <*0.0001), hospitalization in ICU (HR 1.24; 95% CI 1.08–1.43; *p <*0.01), mechanical ventilation (HR 1.50; 95% CI 1.25–1.81; *p <*0.0001), and time period 2017-2018 (HR 0.74; 95% CI 0.65–0.83; *p <*0.0001) (Table 5). When analyses were restricted to the CLD-ICU group, multivariable cox-regression analyses identified age (HR 1.03, 95% CI 1.02–1.04; *p <*0.0001) (Table 4), mechanical ventilation (HR 1.57, 95% CI 1.25–1.97; *p <*0.001) and French donor risk score (HR 1.05; 95% CI 1.02–1.09; *p <*0.001) as associated with 5-year mortality (Table 6), while period of LT was not.

**Table 5:** Predictors of 5-year mortality after LT in the overall CLD cohort.

Variables	Univariate models			Multivariate models		
	HR	95% CI	*p* value	HR	95% CI	*p* value
Age at LT (per year)	1.03	1.03–1.03	<0.0001	1.03	1.03–1.04	<0.0001
MELD (per point)	1.0	0.999–1.01	0.103	1	0.97–1.01	0.578
Mechanical ventilation	1.67	1.46–1.9	<0.0001	1.5	1.25–1.81	0.0001
Renal replacement therapy	1.59	1.35–1.86	<0.0001	1.34	0.873–2.06	0.18
ICU status at LT	1.38	1.26–1.51	<0.0001	1.24	1.08 –1.43	0.00256
LT time period *vs.* 2008-2010						
2017-2018	0.77	0.69–0.86	0.0001	0.736	0.651–0.83	0.0001
2014-2016	0.69	0.61–1.01	0.05	0.98	0.69–1.4	0.94
2011-2013	0.85	0.67–1.09	0.21	1.09	0.78–1.52	0.6

CLD, chronic liver disease; HR, hazard ratio; ICU, intensive care unit; LT, liver transplantation; MELD, model for end-stage liver disease.

ICU, intensive care unit.

Data are expressed as number (%). *p* values are the results of chi^2^ test for qualitative variables *p* <0.05 is significant.

**Table 6:** Predictors of 5-year mortality after LT in CLD-ICU patients.

Variables	Univariate models			Multivariate models		
	HR	95% CI	*p* value	HR	95% CI	*p* value
Age at LT (per year)	1.03	1.02–1.03	<0.0001	1.03	1.02–1.04	<0.0001
MELD (per point)	1.0	0.99–1.01	0.4	1.01	0.99–1.02	0.359
Mechanical ventilation	1.46	1.22–1.74	<0.0001	1.57	1.25–1.97	0.0001
Bilirubin (μmol/L) (per point)	1	1–1.01	0.63			
Creatinine (μmol/L) (per point)	1	1–1.01	0.19			
INR (per point)	0.94	0.93–1.06	0.84			
French donor risk score (risk for each 1-point increase)	1.05	1.02–1.08	0.002	1.05	1.02 –1.09	0.001
Renal replacement therapy	1.3	1.07–1.5	<0.0001	1.27	0.72; 2.22	0.40
LT time period *vs.* 2008-2010 (reference)						
2017-2018	0.83	0.62–1.1	0.1	1.02	0.69–1.49	0.91
2014-2016	0.78	0.61–1.01	0.05	0.98	0.69–1.4	0.94
2011-2013	0.85	0.67–1.09	0.21	1.09	0.78–1.52	0.6
Time period of CLD-ICU LT 2008-2010 *vs*. 2011-2018	1.21	0.97–1.5	0.083			
Year of liver transplant since 2008 (risk for each 1-year increase)	1	0.97–1.03	0.93			

CLD, chronic liver disease; HR, hazard ratio; ICU, intensive care unit; INR, international normalized ratio; LT, liver transplantation; MELD, model for end-stage liver disease.

Considering the impact of age on outcomes, we stratified the cohort using two age thresholds: 50 and 60 years. Compared with recipients ≤50 years, those aged 50–60 years had an increased risk of death (HR 1.66; 95% CI 1.50–1.84; *p* <0.0001), and those aged 60–74 years had approximately a twofold higher risk (HR 2.00; 95% CI 1.80–2.21; *p* <0.0001) (Fig. S5).

Furthermore, multivariable cox-regression analyses restricted to the first year after LT in the CLD-ICU group identified the same variables – age (HR 1.03; 95% CI 1.01–1.04; *p <*0.0001), mechanical ventilation (HR 1.63; 95% CI 1.2–2.04; *p <*0.001), and French donor risk score (HR 1.06; 95% CI 1.02–1.1; *p =* 0.003) – as associated with 1-year mortality (Table S4).

## Discussion

Our study shows that 5-year survival rates of patients transplanted while in the ICU were significantly lower compared to non-ICU patients. Regarding temporal trends, we observed that the volume of LT performed for ICU patients increased over time, but we did not observe an improvement in 5-year survival rates over time.

While outcomes for patients undergoing LT while in the ICU in expert centers are favorable, these patients still experience a significantly worse prognosis compared to those who are not critically ill at the time of transplantation.^9^^,^^12^ Our nationwide French study confirms this disparity, with a 5-year survival rate of 69.1% for patients transplanted while in the ICU, which is significantly lower than the 77.8% observed in the non-ICU cohort (95% CI 76.9–78.6, *p <*0.001). Nonetheless, this survival rate seems acceptable, as it approaches the upper limit (70%) of the thresholds often utilized to justify utility in the field of LT.^14^^,^^21^ The 5-year survival rate in our nationwide cohort is also similar to the 72.6% observed in a cohort consisting of highly selected ACLF-3 patients transplanted in expert centers^13^^,^^22^ and the 66.7% reported in a cohort of ACLF-3 patients from the United Network for Organ Sharing (UNOS) database.^12^

Interestingly, although overall 5-year survival was significantly lower in the ICU group compared to non-ICU patients, this difference was driven by early post-transplant mortality. As shown in our results, 56.3% of deaths in the ICU group occurred within the first 2 years, with nearly half occurring within the first year. However, when analyzing conditional survival among patients who survived the first year, outcomes were similar between the two groups. This finding suggests that the early perioperative period is the main driver of the survival gap.

In line with our findings, a recent study, using data from the UNOS database, reported a persistent survival gap between critically ill and non-critically ill patients with cirrhosis, although this gap reduced over time.^15^ In this study, the 5-year survival rate for critically ill recipients improved from 65.2% (2009–2012) to 74.2% (2013–2016).^15^ In our cohort, a relative increase in 5-year survival was also observed – from 65.6% in 2008–2010 to 68.7% in 2017–2018 – but this improvement was not statistically significant. We also observed an increase in the proportion of patients undergoing LT while in the ICU, along with evolving patient and donor characteristics. However, despite these trends, no clear improvement in survival emerged that would suggest a learning curve effect. Several factors may explain this: our sample was smaller compared to the UNOS cohort (1,291 *vs.* 5,800 critically ill patients) and our observation period was shorter (11 years *vs.* 16 years in the US study, which extended up to 2020). Finally, donor profiles differed significantly between the two cohorts. While donor age in the US decreased from 41 to 36 years (2005–2020), donor age in France increased from 50.9 years in 2008 to 57.5 years in 2018,^23^ potentially impacting outcomes, particularly in this critically ill study population. Our findings indicate a progressive decline in donor quality over time, reflected not only by increasing donor age but also by higher French donor risk scores among CLD-ICU recipients. This score incorporates key variables such as diabetes, hypertension, and vascular cause of death, which increased in the donor population during the study period. In our study, the French donor risk score was independently associated with both 1- and 5-year mortality in CLD-ICU patients, underscoring the prognostic relevance of donor quality in this high-risk group. These findings suggest that, despite recipient severity, graft-related factors remain critical determinants of long-term outcomes after ICU transplantation. To our knowledge, this is the first study to report such an association in a national cohort, and these findings warrant external validation in other populations and transplant systems.

However, the present study does not demonstrate a significant improvement in long-term prognosis following LT in the CLD-ICU group, underscoring the need to optimize both donor and candidate selection as well as post-transplant management.

Recipient age emerged as a significant prognostic factor in this CLD-ICU group. Specifically, recipients aged >60 years exhibited a twofold increased risk of mortality compared to those aged <50 years (HR = 2.00; 95% CI 1.80–2.21; *p* <0.0001). Lerosey *et al.*^24^ also showed that recipient age significantly influences post-LT survival, with a strong effect within the first 2 years after transplantation. Progress depends on the successful application of recently developed scoring systems to better identify suitable candidates and define the optimal transplantation window,^25^^,^^26^ as well as the accumulating evidence highlighting key variables to consider when selecting patients for LT.^27^ However, the lack of prognostic improvement after the first post-transplant year is particularly concerning. In the present study, as in other studies exploring long-term outcomes in this population, the leading causes of 5-year death were infections (22.7%) and cardiovascular events (12.8%).^12^

Infection and multi-organ failure also emerged as the leading causes of early post-LT mortality among CLD-ICU recipients, jointly accounting for over 50% of deaths within the first 3 months. Notably, in the most recent period (2017–2018), infections represented 27.6% of late deaths at 5 years, nearly twice the rate of cardiovascular-related deaths (13.3%). The observed association between ICU status and infection-related mortality highlights a critical area for intervention, particularly during the early (<3 months) post-LT period. With an odds ratio of 1.55 (95% CI 1.05–2.29; *p* = 0.02), ICU admission appears to be an independent risk factor for infection-related mortality, supporting the need for clinical protocols aimed at reducing infectious complications in this high-risk population. Moreover, the implementation of tailored immunosuppression strategies to reduce infectious complications, along with enhanced pre-transplant evaluation and post-transplant management of cardiovascular comorbidities, is essential to improve outcomes in this high-risk population. As for the challenge of conducting comprehensive cardiovascular assessments in bedridden ICU patients, an important step forward could be the use of dedicated risk stratification tools, such as the CAD-LT score,^28^ to guide cardiovascular evaluation.

Our findings align with a recent large-scale US registry study, which used a marginal structural model to adjust for waitlist selection bias. They found that ACLF-3 at the time of transplant was independently associated with a significantly higher risk of 1-year post-LT mortality, but not with increased long-term mortality beyond 1 year. Their results reinforce the importance of transplant timing and strengthen the rationale for early identification and individualized risk stratification to improve survival in critically ill LT candidates.^29^

Our study supports several practical recommendations for managing high-risk LT candidates in the ICU. Given the clear impact of age on survival, especially above 60 years, there is a need to consider careful selection of older candidates. While age should not be a strict cut-off, it should trigger multidisciplinary evaluation.

The independent effect of donor quality suggests that optimal grafts should be prioritized for critically ill patients. When feasible, ICU patients, especially those intubated or with multiple organ failures, may benefit from optimal grafts, given their already elevated baseline risk. Donor-recipient matching in ICU patients should avoid compounding risks. Tailored donor-recipient matching, factoring both clinical severity and graft risk, could improve outcomes. Finally, the high rate of infection-related deaths highlights the need for early infectious disease consultation and proactive prevention protocols in all ICU recipients during the perioperative period.

This study has several limitations, primarily due to its retrospective design and reliance on registry data, despite the prospective collection of some variables. In particular, detailed information on the pre-transplant period such as ICU length of stay and clinical events prior to LT was unavailable. Critical variables such as pre-existing comorbidities, infections, cardiovascular disease or tobacco use, were not available, which may have influenced both selection for transplantation and post-transplant outcomes. Moreover, heterogeneity among ICU patients, combined with the lack of granular data on organ failures as defined by EASL CLIF-OF criteria, may have limited our ability to stratify patients by severity and to detect significant differences in survival trends over time.

Our study also has several strengths, notably the large scale of our cohort, which encompasses all LTs performed in France during the study period, and the reliability of the CRISTAL database, which benefits from prospective data collection.

In conclusion, although patients with CLD undergoing LT from the ICU have lower long-term survival compared to non-ICU recipients, their 5-year outcomes remain within an acceptable range, approaching the upper thresholds commonly used to define organ utility. Contrary to prior reports, we did not observe a significant improvement in post-transplant prognosis following LT in this population. This may be partly explained by the increasingly frequent allocation of poorer-quality grafts to these patients over time, which could have mitigated potential gains in outcome. These results underscore the importance of optimizing perioperative management and graft quality, implementing targeted prevention of infections and cardiovascular events, and further refining candidate selection in order to improve outcomes in this high-risk group.

## Abbreviations

ACLF, acute-on-chronic liver failure; CLD, chronic liver disease; CLD-ICU, chronic liver disease patients in the intensive care unit; HR, hazard ratio; ICU, intensive care unit; LT, liver transplantation; OF, organ failure; RRT, renal replacement therapy; UNOS, United Network for Organ Sharing.

## Ethics approval and consent to participate

The ethics committee of the University Hospital of Montpellier granted ethical approval. Number 2019-IRB-MTP_09-13. The research committee of Agence de la Biomedicine granted approval.

## Authors’ contributions

All authors contributed to data interpretation and reviewed, revised, and approved the manuscript. Drs Meszaros, Ursic Bedoya, Artru and Pageaux had full access to all the data in the study and take responsibility for the integrity and the accuracy of the data analysis. Concept and design: Drs Meszaros, Ursic-Bedoya, Artru, Pageaux, Dumortier, Dharancy. Statistical analysis: Dr Meszaros, Artru and Ursic-Bedoya.

## Data availability

Data are available from the French ABM. Access requests must be submitted to the French ABM and approved by the French ABM, in accordance with national regulations.

## Declaration of generative AI and AI-assisted technologies in the writing process

During the preparation of this work, the authors used ChatGPT 4o to improve readability. After using this tool/service, the authors reviewed and edited the content as needed and take full responsibility for the content of the publication.

## Financial support

No financial support was received to produce this manuscript.

## Conflict of interests

The authors declare that they have no competing interests.

Please refer to the accompanying ICMJE disclosure forms for further details.
