# Supplementary material for: Long-term outcomes and temporal trends following liver transplantation for chronic liver disease in the intensive care unit
Source: JHEP Rep. 2026 Jan 8;8(4):101722. doi: 10.1016/j.jhepr.2025.101722 (PMC12969417; doi:10.1016/j.jhepr.2025.101722)

Long-term outcomes and temporal trends following liver transplantation for chronic liver disease in the intensive care unit

Magdalena Meszaros, José Ursic-Bedoya, Audrey Coilly, Claire Francoz, Cristophe. Duvoux, Filomena Conti, Francois Faitot, Pauline Houssel-Debry, Jean Hardwigsen, Marie-Noelle Hilleret, Claire Vanlemmens, Laure Elkrief, Nassim Kamar, Rodolphe Anty, Armand Abergel, Claire Perignon, Laurence Chiche, Maryline Debette-Gratien, Teresa Antonini, Corinne Antoine, Sebastien Dharancy, Jérome Dumortier, Georges Philippe Pageaux, Florent Artru

Table of contents

Table S1……………………………………………………………………………….....2

Table S2……………………………………………………………………………....…3

Table S3 ……………………………………………………………………………......4

Table S4……………………………………………………………………….…………6

Fig. S1……………………………………………………………………………………7

Fig. S2……………………………………………………………………………………8

Fig. S3……………………………………………………………………………………9

Fig. S4…………………………………………………………………………………..10

Fig. S5…………………………………………………………………………………..11

Table S1:

Patient characteristics of the overall CLD at the time of LT (N=9,763)

**Characteristics**	**N=9,763**
**Age et LT (years) ***	57 (50.6)
**Sex (male), %**	7,418 (76%)
**BMI Mea, kg/m2**	26.5 (6.1)
**Liver disease requiring LT, n (%)**	
HCC	3,382 (34.6%)
**Alcohol related Liver Disease (ALD)**	3,225 (33.0%)
Viral hepatitis	1158 (11.9%)
Others	1157 (11.9%)
Autoimmune or Cholestatic	841 (8.6%)
**Medical Status at LT, n (%)**	
Home	7169 (74%)
ICU	1291 (13.3%)
Liver ward	1232 (12.7%)
**Biological Parameters at LT**	
Bilirubin, (µmol/L)	117.5 (181.4%)
**Creatinine (µmol/L), mean (SD)**	85.44 (45.7%)
**Sodium (mmol/L), Mean (SD)**	136.48 (5.4%)
**MELD Score at LT , Mean (SD)**	15.06 (10.0%)
**Mechanical ventilation, n (%)**	487 (5.4%)
**Renal replacement therapy**	333 (3.5%)
**Number of organ failures***	
1	1731
2	802
3 or more	484
**Immunosuppression regimen after LT°**	
Corticosteroids	7959 (81.5%)
Tacrolimus	7256 (74.3%)
Mycophenolic acid	7790 (79.8%)
Azathioprine	20 (0.2%)
Induction using anti lymphocyte Ab	211 (2.2%)

Abbreviations: ALD, alcohol-associated liver disease; Ab antibodies, BMI, body mass index;

INR, international normalized ratio; ICU, intensive care unit; LT, liver transplantation; MELD, Model for End-stage Liver Disease score; was expressed in mean (SD) or n (%) unless* median (IQR).

*exclusion of brain and circulatory failure as non-collected in CRISTAL database; ° closest to liver transplantation

Other: polycystic liver disease, and rare genetic/metabolic diseases , vascular liver disease, MASLD

Table S2 : Causes of 3-months mortality in CLD-ICU and non-ICU patients

**Causes of death**	**CLD ICU (n= 170)**	**CLD non ICU (n=391)**
Sepsis	**52 (30.6%)**	**73 (18.7%)**
Multi-organ failure	**36 (21.2%)**	**65 (16.6%)**
Cardio vascular	**35 (20.6%)**	**85 (21.7%)**
Other specified complication	**11 (6.5%)**	**64 (16.4%)**
Surgical hemorrhage	**11 (6.5%)**	**42 (10.7%)**
Primary graft failure	**5 (2.9%)**	**10 (2.6%)**
Recurrence of initial disease	**5 (2.9%)**	**2 (0.5%)**
Graft vascular complications	**4 (2.3)%**	**18 (4.6%)**
Other graft-related complications	**2 (1.2%)**	**3 (0.8%)**
Liver failure	**3 (1.7%)**	**9 (2.3%)**
Intraoperative death	**2 (1.2%)**	**4 (1%)**
Acute rejection	**2 (1.2%)**	**2 (0.5%)**
Acute renal failure	**1 (0.6%)**	**1 (0.3%)**
Other unidentified cause of death	**1 (0.6%)**	**13 (3.3%)**

Abbreviations: ICU, intensive care unit;

Table S3 : Temporal trends in characteristics of CLD patients undergoing LT.

**Variable**	**2008-2010 (N=2229)**	**2011-2013 (N=2571)**	**2014-2016 (N=2987)**	**2017-2018 (N=1976)**	**P-Value**
**Sex (male)**	1667 (74.8%)	1969 (76.6%)	2287 (76.6%)	1495 (75.7%)	0.403
**Age at LT (years), Mean (SD)**	53.8 (± 9.5)	54.3(± 9.8)	55.4 (± 10.1)	56.1 (± 10.5)	<0.001
**Initial Disease requiring LT, n (%)**					<0.001
ALD	697 (31.3%)	926 (36.0 %)	987 (33.0%)	615 (31.1%)	
Viral B/C	78 (3.5%)/316 (14.2%)	67(2.6%)/276 (10.7%)	67 (2.2%)/ 224 (7.5%)	58 (2.9%)/ 72 (3.6%)	
Auto-immune, cholestatic	188 (8.4%)	221 (8.6%)	268 (9%)	187 (9.5%)	
Others	950 (42.6%)	1081 (42.0%)	1441 (48.2)	1044 (52.8%)	
**HCC status as initial disease**					<0.001
HCC	728 (32.7%)	830 (32.3%)	1114 (37.3)	779 (39.4%)	
No HCC	1501 (67.3%)	1741 (67.7%)	1873 (62.7%)	1197 (60.6%)	
**Biological Parameters at LT**					
**Bilirubin (µmol/L), Mean (SD)**	103.9 (± 153.4)	128 (± 171.1)	120.6 (± 218.4)	114.64 (± 159.3)	<0.001
INR, Mean (SD)	1.8 (± 1.0)	2.01 (± 1.5)	1.93 (± 1.3)	1.85 (± 1.1)	<0.001
**Creatinine (µmol/L), Mean (SD)**	84.64 (± 43.7)	85.86 (± 45.7)	85.91 (± 48.2)	85.12 (± 44.5)	0.257
**MELD Score at LT Mean (SD)**	14.74 (± 9.3)	15.9 (± 10.4)	14.89 (± 10.2)	14.55 (± 9.9)	<0.001
**Mechanical Ventilation**	78 (4.0%)	166 (7.2%)	148 (5.1%)	95 (5%)	<0.001
**Renal Replacement Therapy**	63 (3%)	80 (3.2%)	115 (3.9%)	75 (3.8%)	0.193
**Liver Failure**	321 (14.4%)	508 (19.8%)	553 (18.5)	335 (17%)	<0.001
**Renal Failure**	124 (5.6%)	205 (8%)	257 (8.6%)	148 (7.5%)	<0.001

**Coagulation Failure**	316 (14.2%)	507 (19.7%)	507 (17%)	328 (16.6%)	<0.001
**ICU Admission**	192 (8.6%)	384 (14.9%)	424 (14.2%)	291(14.7)	<0.001

Abbreviations: LT, liver transplantation; CLD, chronic liver disease; ALD, alcohol-related liver disease; HCC, hepatocellular carcinoma; RRT, renal replacement therapy; INR, international normalized ratio; MELD, Model for End-Stage Liver Disease; SD, standard deviation; ICU, intensive care unit.

Table S4: Predictors of 1-year mortality post LT in CLD-ICU patients

Variables	Univariate models			Multivariate models		
	HR	95%CI	P value	HR	95%CI	P value
Age at LT (per year)	1.02	1.01 - 1.03	<0.0001	1.03	1.01-1.04	<0.0001
MELD (per point)	1	0.99 - 1.01	0.74	1.01	0.99-1.01	0.572
Mechanical ventilation	1.46	1.22 - 1.75	<0.0001	1.63	1.3-2.04	0.0001
Bilirubin (µmol/L),(per point)	1	1-1.01	0.63			
Creatinine (µmol/L) (per point)	1	1-1.01	0.19			
INR (per point)	0.99	0.93-1.06	0.84			
French Donor Risk Score (risk for each 1 point increase)	1.05	1.02-1.08	0.02	1.06	1.02-1.1	0.003
Renal replacement therapy	1.26	1.04-1.53	<0.0001	1.11	0.63 ; 1.95	0.7
LT Time period<br>2017-2018<br>2014-2016<br>2011-2013<br><br>vs. 2008-2010 (reference)	 1.05<br>0.95<br>0.94	 0.78 - 1.4<br>0.73-1.4<br>0.74-1.21	 0.1<br>0.05<br>0.21	 1.3<br>1.21<br>1.2	 0.88-1.92<br>0.84-1.73<br>0.86-1.68	 0.18<br>0.3<br>0.27
Year of liver transplant since 2008<br>(risk for each 1 year increase)	1	0.97-1	0.93			

Fig. S1. (A) Overall 5-year graft survival (B) 5-year graft survival according to ICU status

A

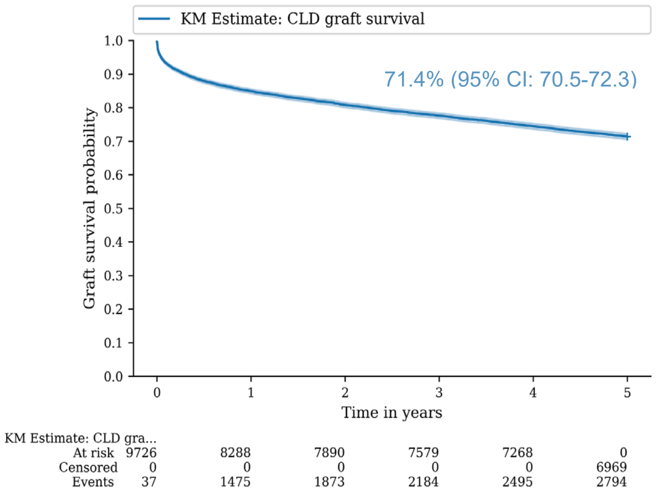

B

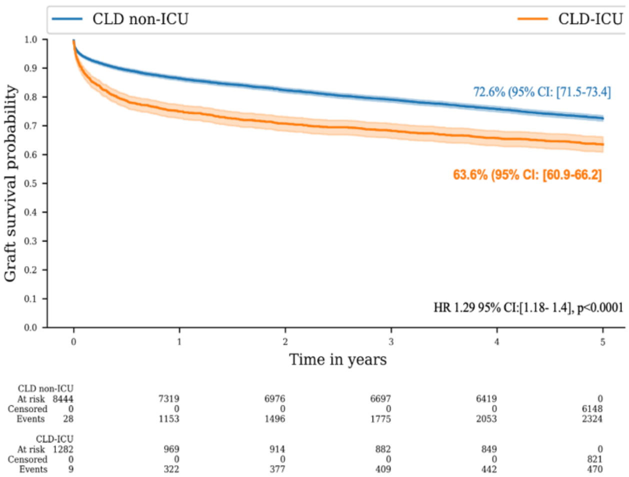

Fig. S2. Evolution of 5-year survival of CLD patients according to time period of LT

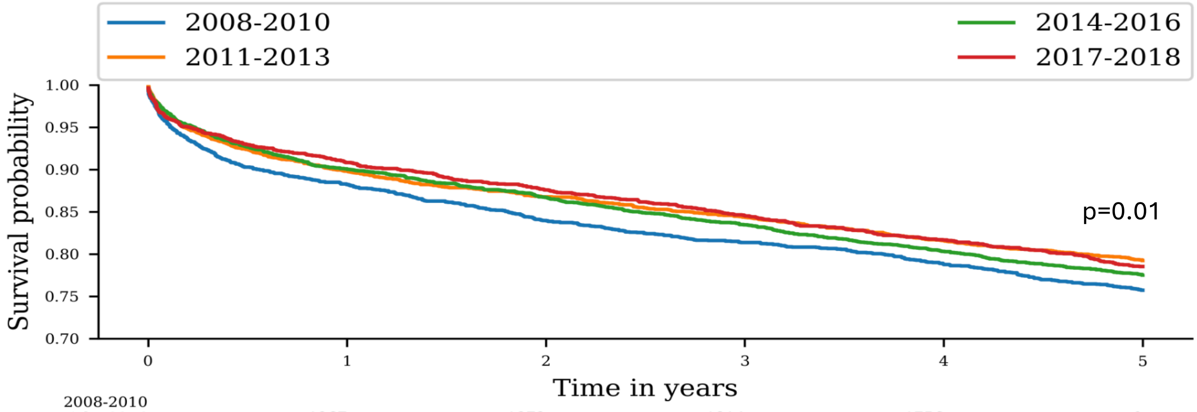

	0	1	2	3	4	5
**2008-2010**						
At risk	2216	1967	1872	1814	1756	0
Censored	0	0	0	0	0	1688
Events	13	262	357	415	473	541
**2011-2013**						
At risk	2567	2309	2231	2170	2098	0
Censored	0	0	0	0	0	2038
Events	4	262	340	401	473	533
**2014-2016**						
At risk	2977	2689	2590	2493	2400	0
Censored	0	0	0	0	0	2316
Events	10	298	397	494	587	671
**2017-2018**						
At risk	1969	1795	1731	1671	1614	0
Censored	0	0	0	0	0	1552
Events	7	181	245	305	362	424

Fig. S3. Evolution of 5-year survival of CLD non-ICU patients according to time period of LT

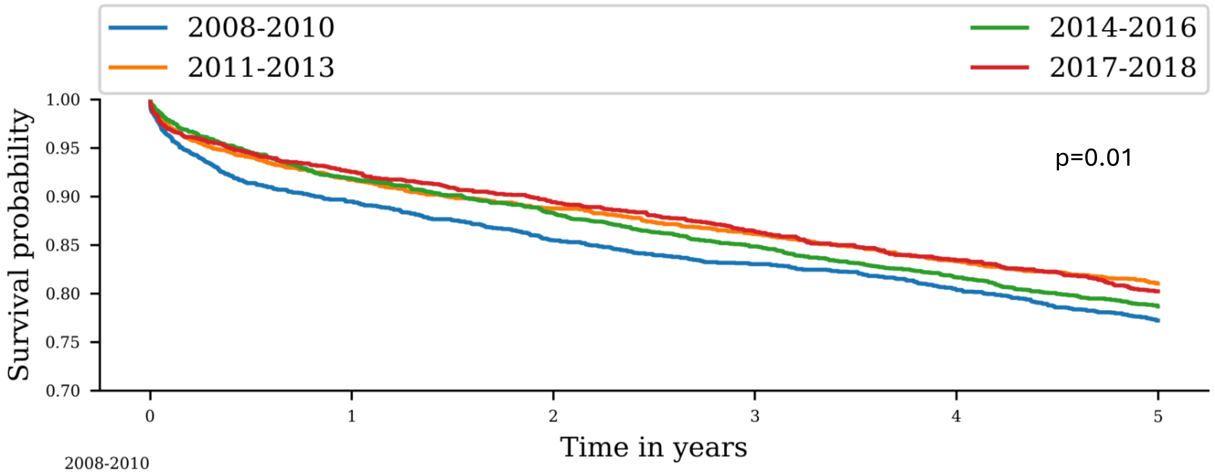

	0	1	2	3	4	5
**2008-2010**						
At risk	1968	1772	1693	1644	1591	0
Censored	0	0	0	0	0	1529
Events	12	208	287	336	389	451
**2011-2013**						
At risk	2172	1994	1930	1873	1812	0
Censored	0	0	0	0	0	1762
Events	2	180	244	301	362	412
**2014-2016**						
At risk	2557	2352	2262	2174	2093	0
Censored	0	0	0	0	0	2016
Events	5	210	300	388	469	546
**2017-2018**						
At risk	1680	1559	1507	1456	1406	0
Censored	0	0	0	0	0	1352
Events	5	126	178	229	279	333

Fig. S4. Evolution of 5-year survival of CLD CU patients according to time-period of LT

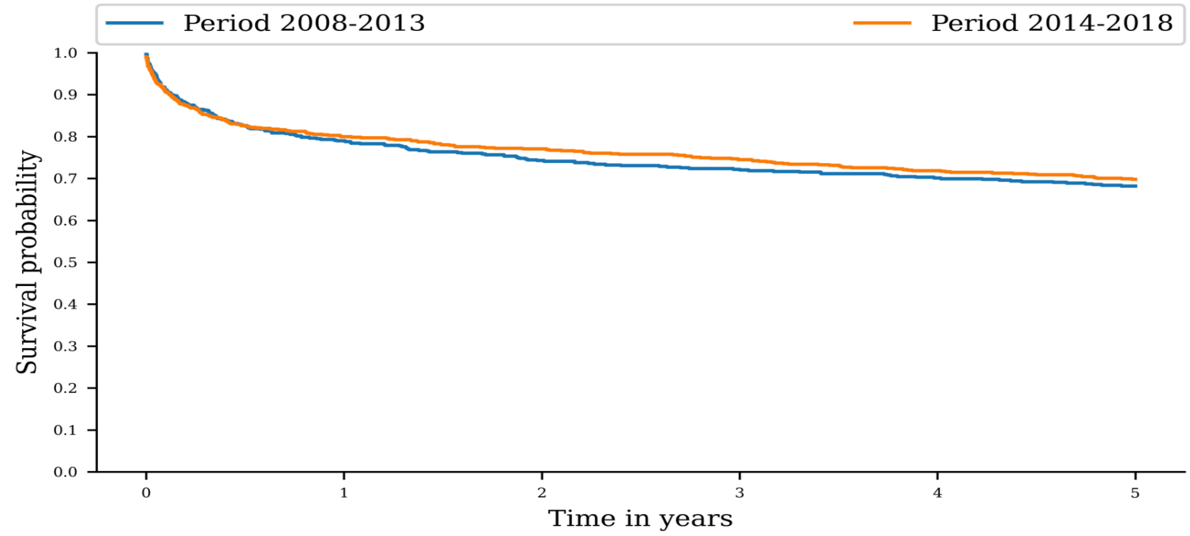

	0	1	2	3	4	5
**Period 2008-2013**						
At risk	574	455	428	416	404	0
Censored	0	0	0	0	0	393
Events	2	121	148	160	172	183
**Period 2014-2018**						
At risk	708	572	551	533	514	0
Censored	0	0	0	0	0	499
Events	7	143	164	182	201	216

Fig. S5. Forest plot of adjusted hazard ratios for predictors of post–liver transplant survival at 1 year (A) and 5 years (B) in CLD ICU patients according to age.

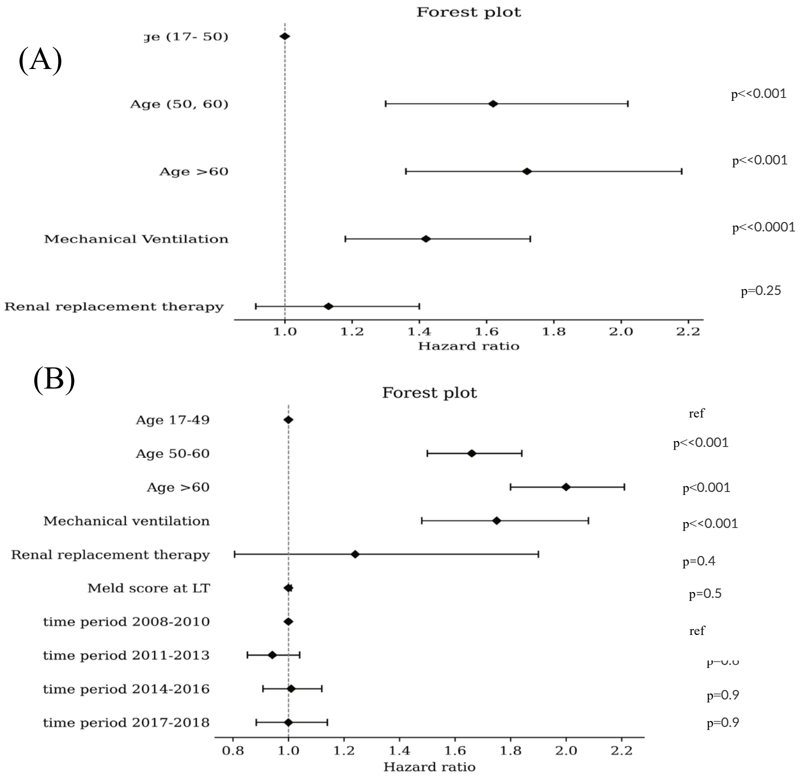

Supplement: Multimedia component 1 [file mmc1.pdf]
